# Supplementary material for: GRK2-Dependent HuR Phosphorylation Regulates HIF1α Activation under Hypoxia or Adrenergic Stress
Source: Cancers (Basel). 2020 May 13;12(5):1216. doi: 10.3390/cancers12051216 (PMC7281538; doi:10.3390/cancers12051216)
Supplement: Supplementary file 1 [file cancers-12-01216-s001.pdf]

Supplementary Materials

GRK2-Dependent HuR Phosphorylation Regulates HIF1α Activation under Hypoxia or Adrenergic Stress

Clara Reglero, Vanesa Lafarga, Verónica Rivas, Ángela Albitre, Paula Ramos, Susana R. Berciano, Olga Tapia, María L. Martínez-Chantar, Federico Mayor Jr and Petronila Penela

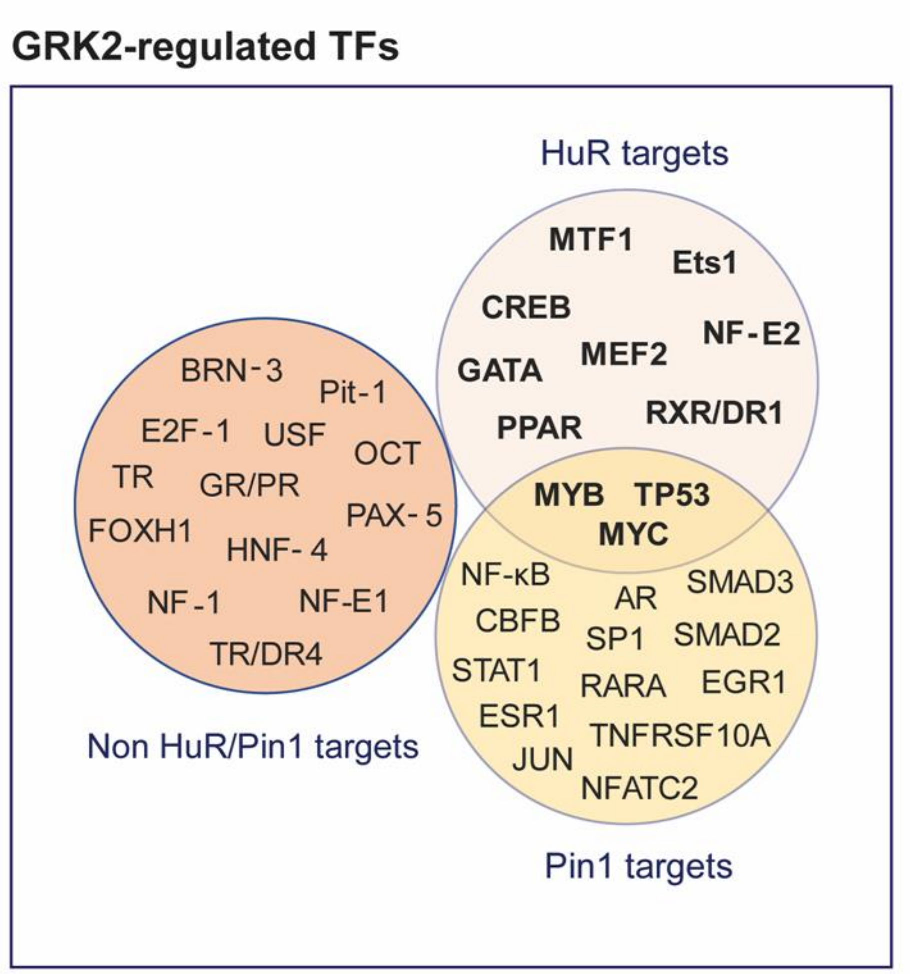

Figure S1. GRK2 expression positively influences the activity of known and predicted HuR-regulated transcription factors in luminal mammary epithelial cells. A reported set of transcription factors involved in cell proliferation, differentiation, transformation or apoptosis, and previously shown to be altered upon stable overexpression of GRK2 non-transformed mammary 184B5 [21], was classified as protein targets of the regulatory functions of either Pin1 or HuR. The regulatory interplay with Pin1 was explored with Ingenuity Pathway Analysis (Qiagen, Hilden, Germany), while the mRNA interaction with HuR was interrogated in a dataset of known and predicted novel mRNA targets derived from different gene data sets analyzed with a U-rich common motif present in experimental HuR-immunoprecipitated mRNAs [27].

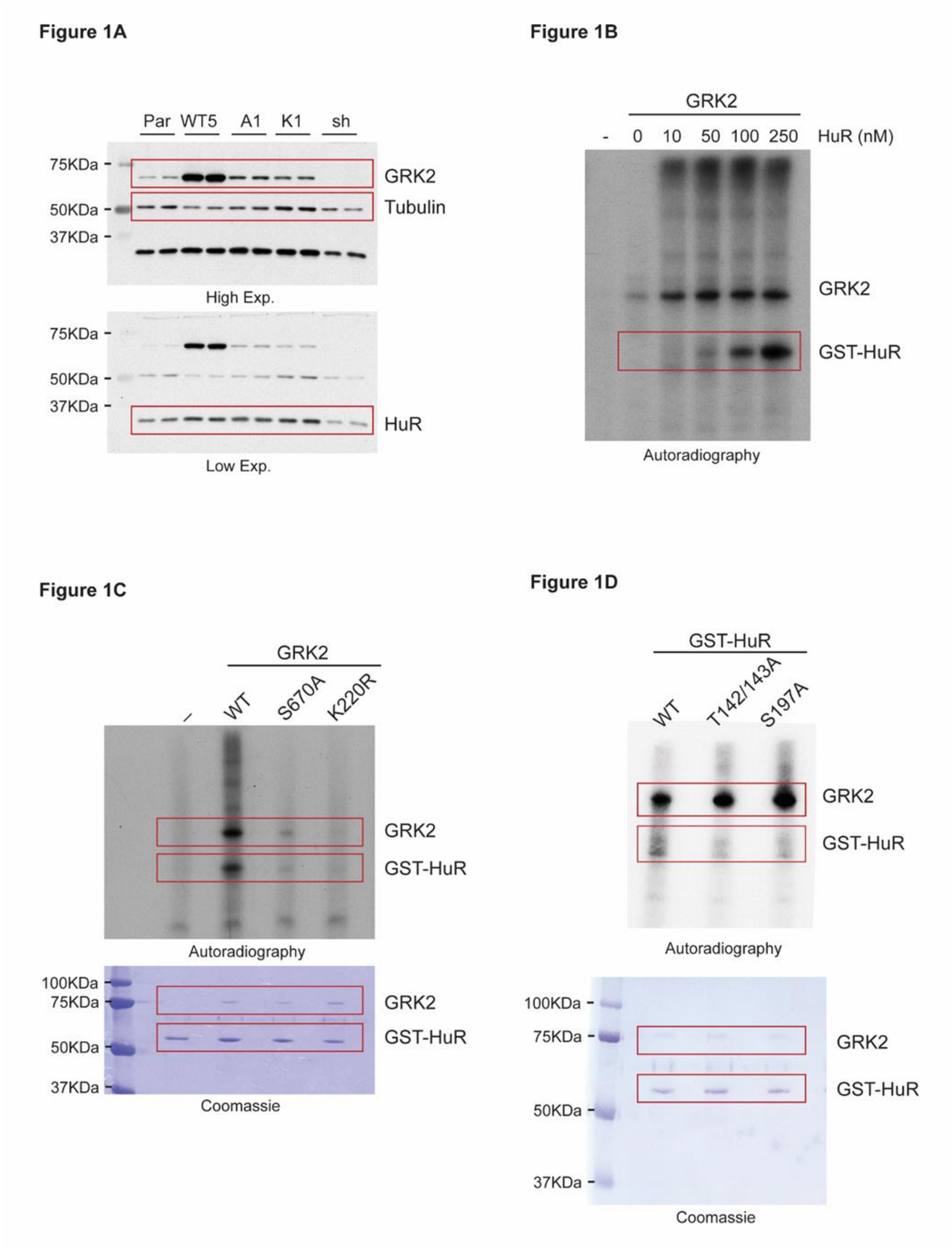

Figure S2. Detailed information about western blot in Figure 1.

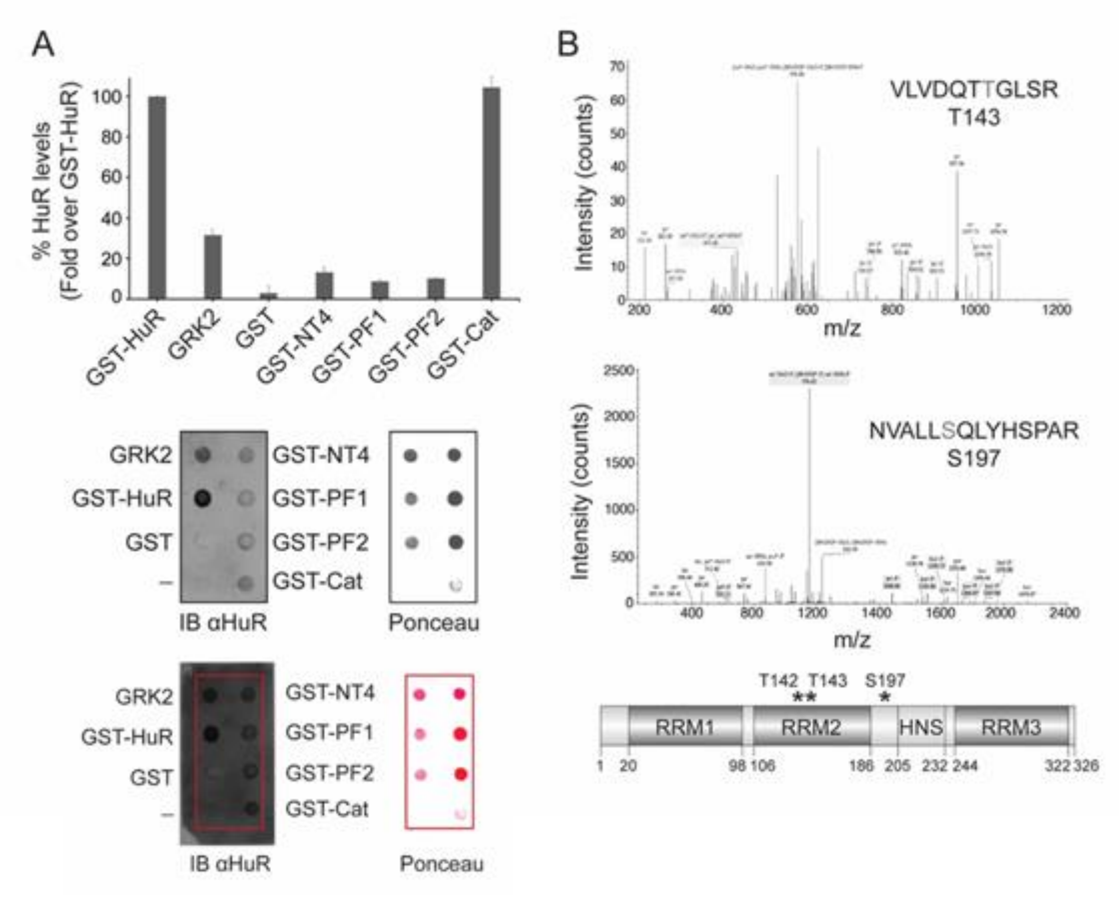

Figure S3. GRK2 binds to and phosphorylates HuR. (**A**) GST-GRK2 fragments and recombinant GRK2 full-length were spotted on strips of nitrocellulose and incubated with GST-HuR. HuR interaction was detected with an anti-HuR antibody. This experiment was performed two times with similar results. (**B**) GRK2 and GST-HuR were incubated in the presence of unlabeled ATP as detailed in Materials and Methods and resolved by SDS-PAGE. The band corresponding to full-length GST-HuR was incubated with trypsin and the resulting peptides were analyzed using LC–MS/MS. Mass spectral analysis of three potential residues is shown, which surrounding sequences displaying acidic amino acids N-terminal to the phosphorylated residue and hydrophilic residues at P + 1, a usual signature of GRK2 phosphorylation sites (Lodowski et al., 2013 [1]). Candidate phosphorylation sites are depicted in the HuR domain structure scheme. Threonine residues 142 and 143 are part of RRM2, the second HuR RNA binding domain, whereas Serine 197 is positioned in the long hinge region (aa 187–243) inter-connecting RRM2 and RRM3 domains, which includes a Nucleocytoplasmic Shuttling (HNS) sequence (aa 205–237) containing both nuclear localization and nuclear export determinants [57] responsible for nuclear/cytoplasmic shuttling of HuR.

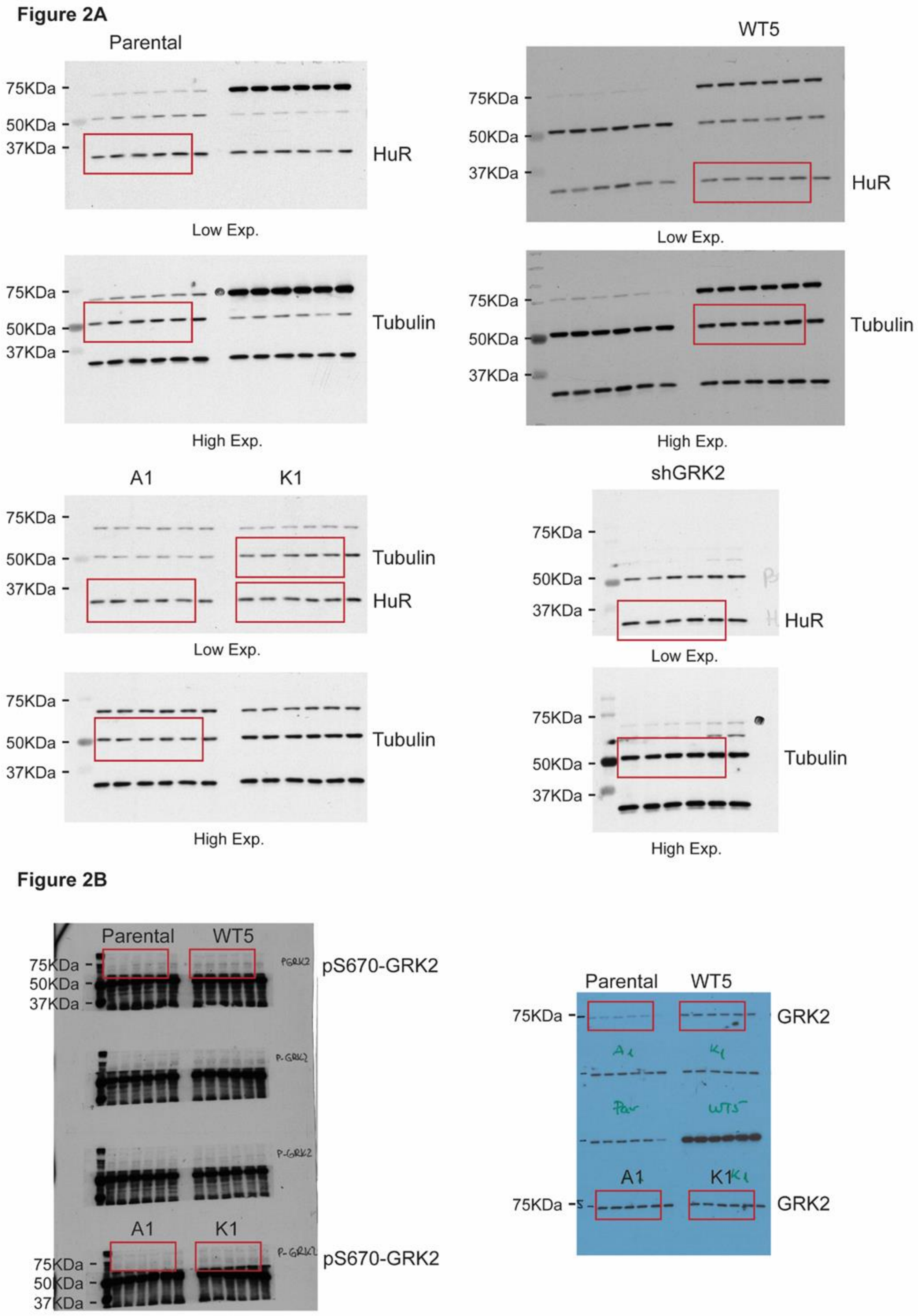

Figure S4. Detailed information about western blot in Figure 2.

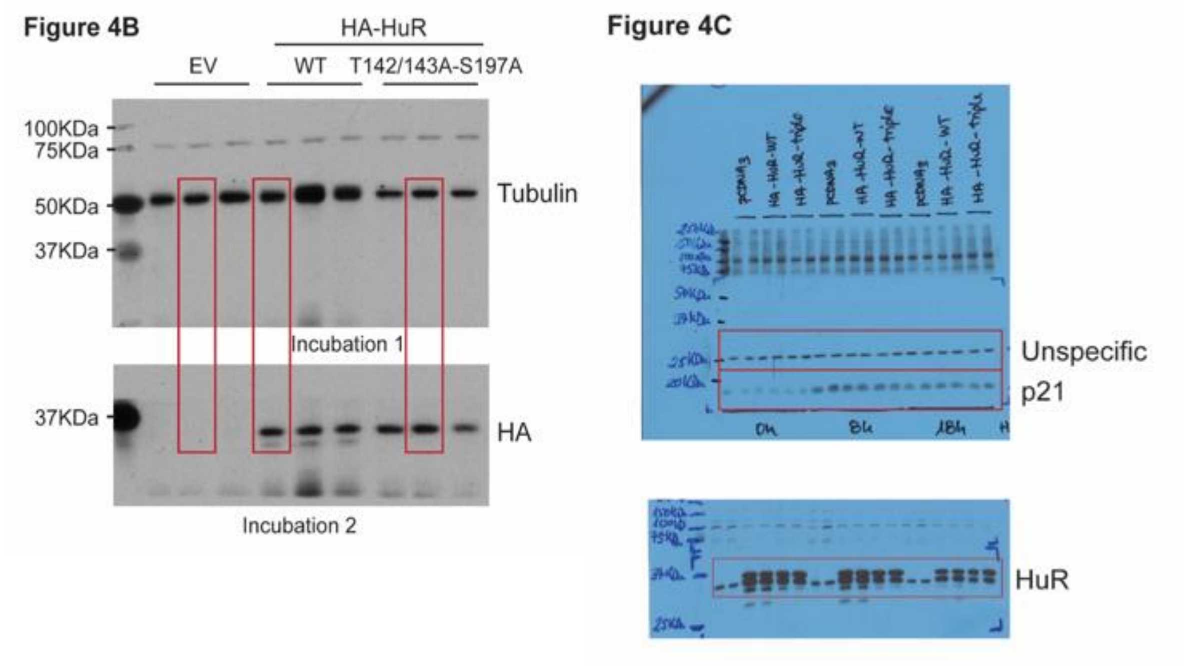

Figure S5. Detailed information about western blot in Figure 4.

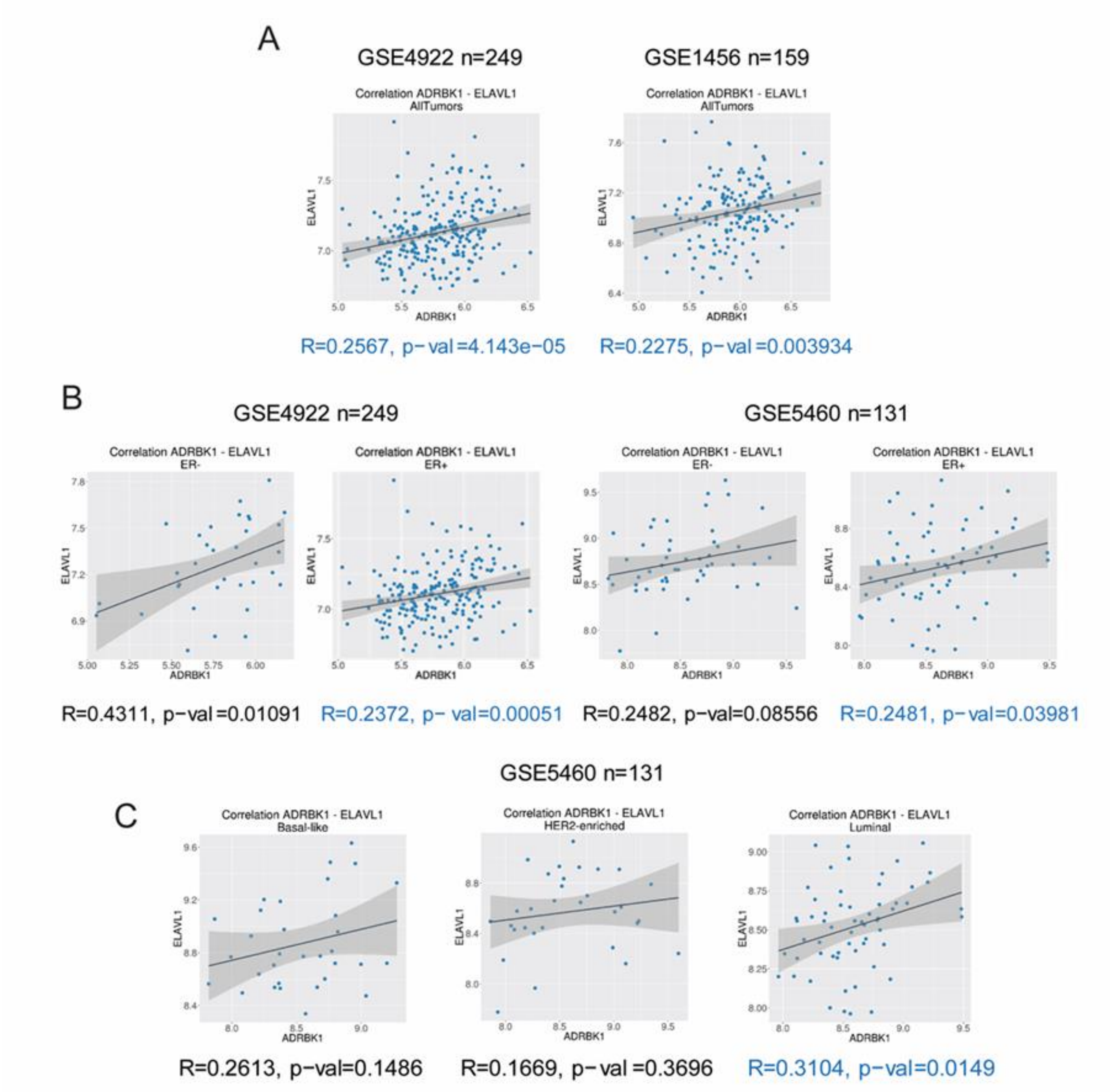

Figure S6. CANCERTOOL analysis of HuR and GRK2 expression in Human Breast Cancers. (**A**) Correlation of expression of *ELAVL1* (HuR) and *ADRBK1* (GRK2) genes in two cohorts of breast cancer (GSE4922 $n = 249$ patient and GSE1456 $n = 159$ patients). Upon analysis of all datasets available (Ivshina (GSE4922), Lu (GSE5460), Metabric (EGAS00000000098), Pawitan (GSE1456), TCGA and Wang (GSE2034)) a direct correlation coherence was found with 60% of the studies presenting a significant correlation ($p < 0.05$) with same directionality. (**B**) Gene correlation in patients stratified according to their estrogen receptor status (GSE4922, $n = 249$ and GSE5460, $n = 131$). (**C**) Correlation analysis of *ELAVL1* and *ADRBK1* gene expression in the molecular subtypes of breast cancer 'basal-like', 'luminal-like' and 'HER2-enriched' across the cohort GSE5460. Pearson correlation coefficient and *p*-value adjusted using Benjamini–Hochberg method are shown. Only direct or inverse correlations with correlation coefficient greater than 20% ($-0.2 < R < 0.2$) and a *p*-value lower than 0.05 were considered.

Figure 5A (Parental vs GRK2)

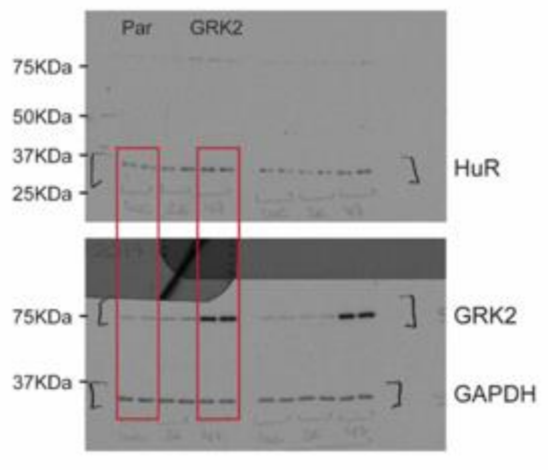

Figure 5A (Parental vs shGRK2)

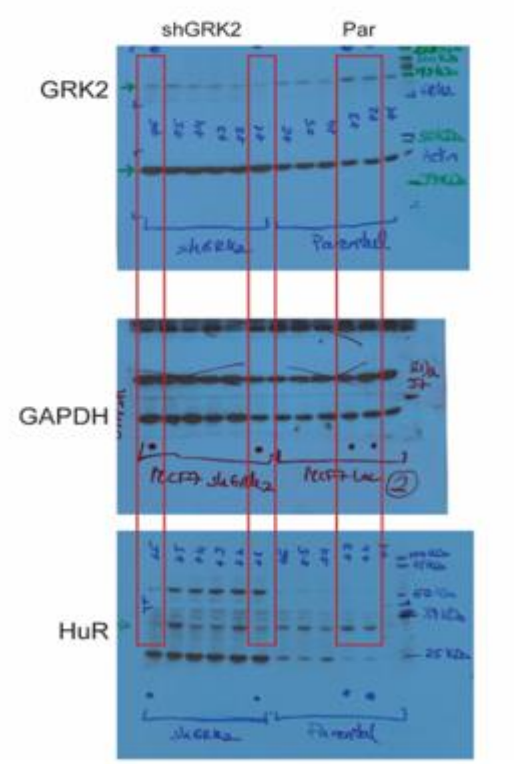

Figure 5B

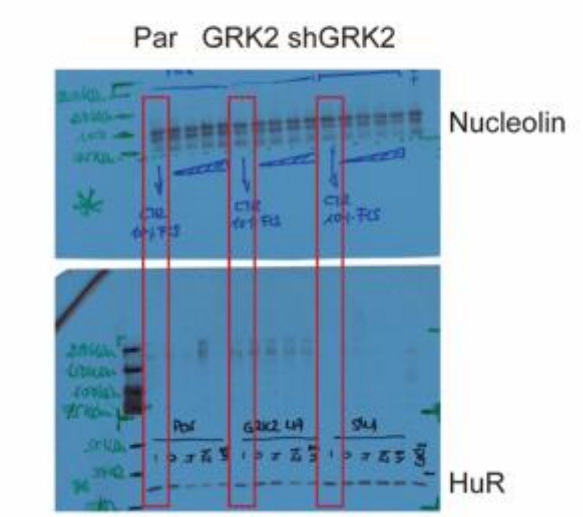

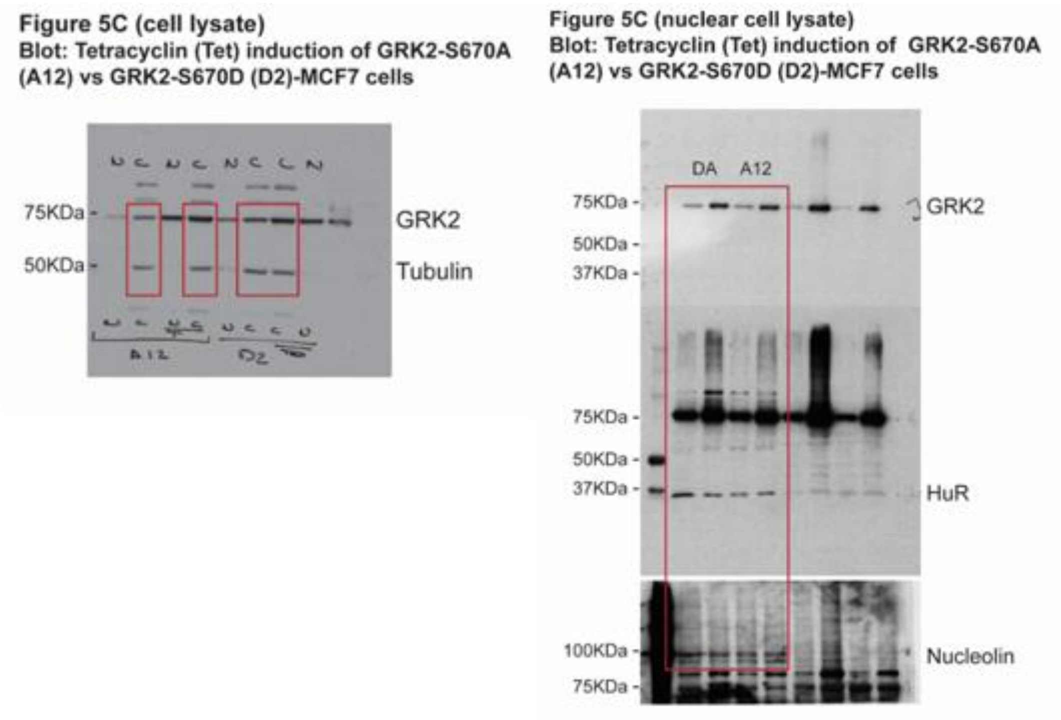

Figure S7. Detailed information about western blot in Figure 5.

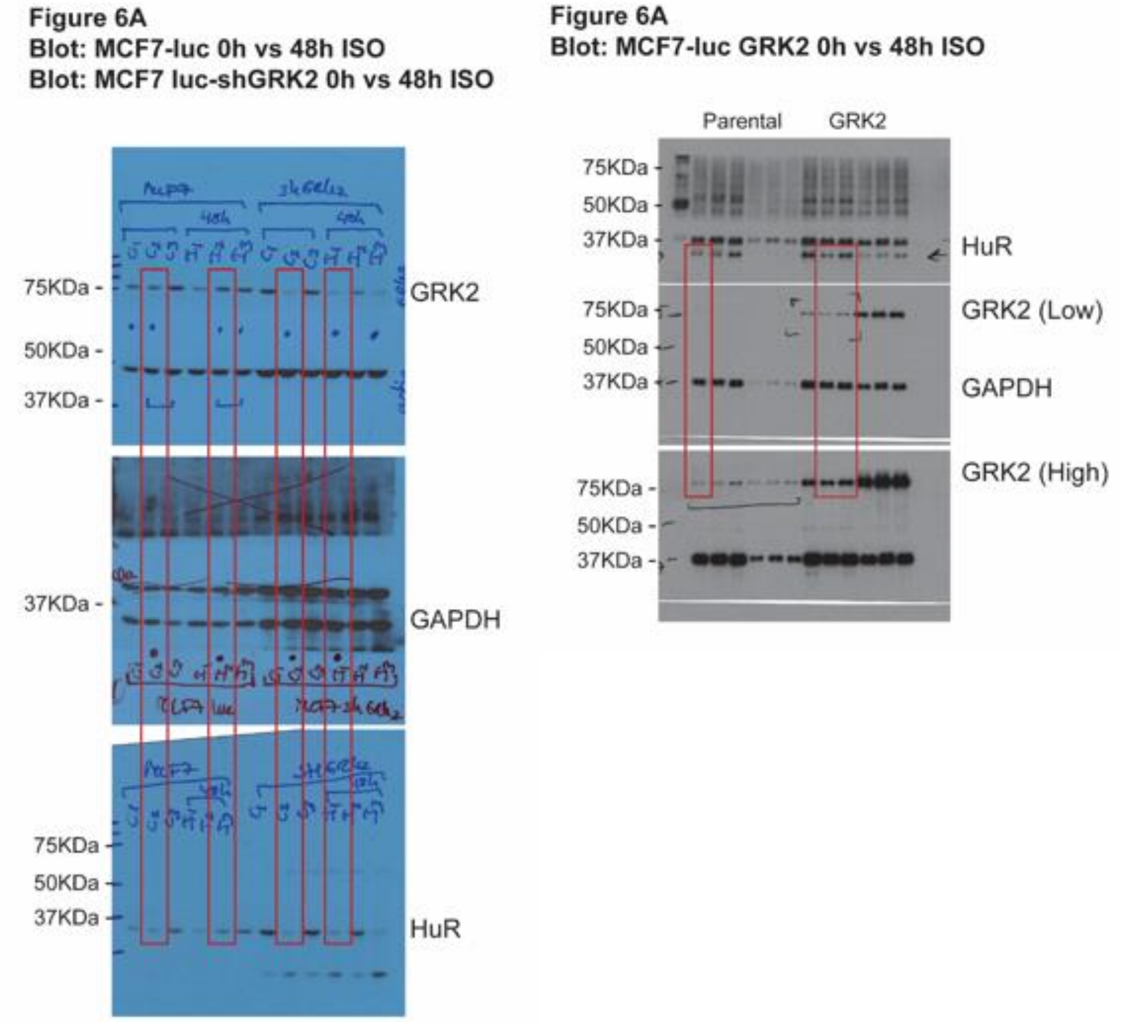

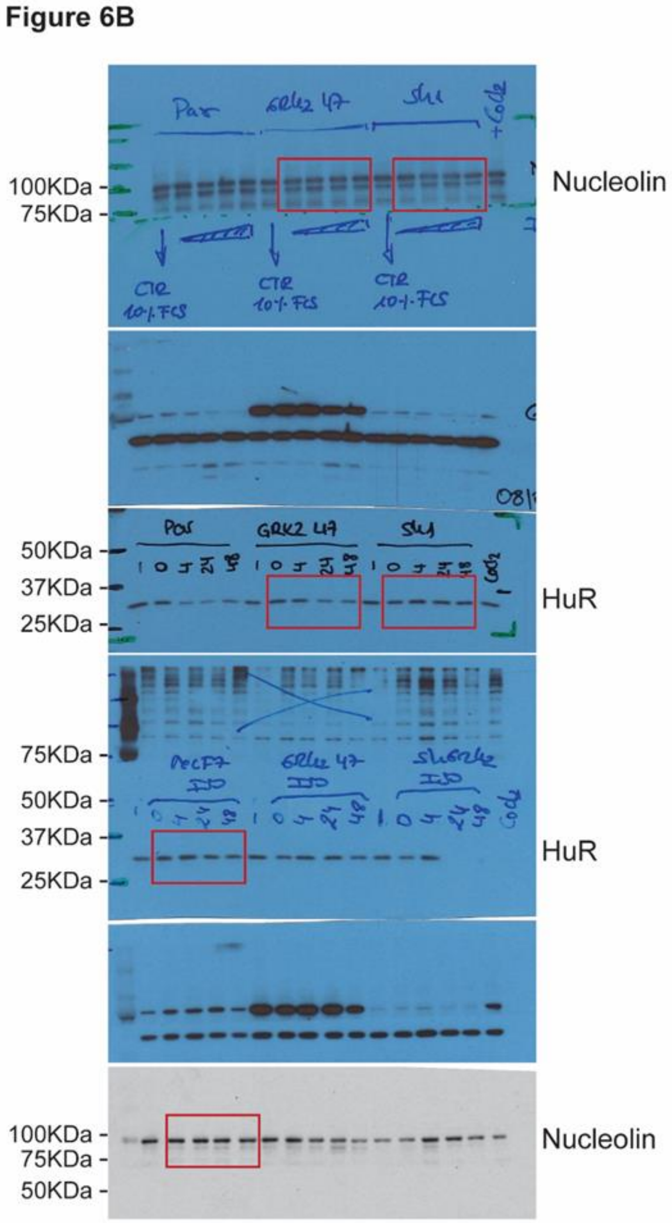

Figure S8. Detailed information about western blot in Figure 6.

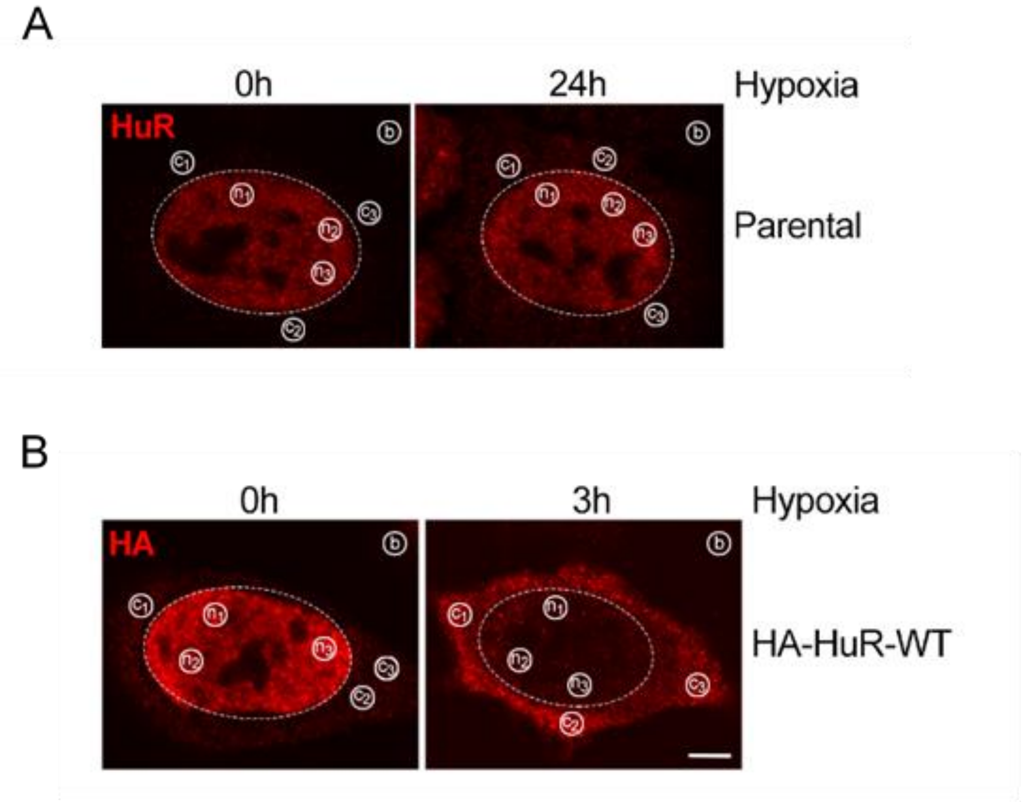

Figure S9. Endogenous HuR or HA-HuR-WT fluorescent image analysis and quantitative data acquisition methodology. The steady-state localization of HuR in Hela cells in basal conditions is clearly nuclear, displaying a well-established and strong signal in the nucleoplasm and being absent in the nucleoli. Although in transformed cell lines robust HuR expression at basal levels is often accompanied by protein accumulation in the cytoplasm, HuR remains predominantly in the nucleus of unstressed Hela cells. Therefore, staining of HuR itself is a reasonable marker for nucleus identification in Hela cells in basal conditions. Stress relocates a portion of HuR to the cytoplasm with a granulated pattern. However, even upon stress a significant amount of HuR remains in the nucleus (easily detected by varying laser strength), making it feasible to exploit such signal as a tool for nuclear identification (Kodiha et al., 2012; Su et al., 2013 [2,3]). To calculate the ratio of cytoplasmic to nuclear fluorescence signal arising from HuR (**A**) or HA (**B**) indirect immunofluorescence in basal or hypoxia-treated cells, nuclear masks (dotted line) were drawn using the nuclear staining distribution pattern of HuR and guided by morphological features (nucleolus, euchromatin, speckles). For each cell, three measurements were made within the nucleoplasm (n1, n2, n3), excluding the HuR-negative areas corresponding to nucleoli and nuclear speckles, or indistinctly in the peripheral cytoplasm (c1, c2, c3). Fluorescence intensity values were corrected for background staining by subtraction of a blank measurement (b) taken outside the cell. The ratio of the mean cytoplasmic (C) to nuclear (N) value was calculated for each cell. Scale bar, 3 μm

Reference

1. Lodowski D.T.; Pitcher J.A.; Capel W.D.; Lefkowitz R.J.; Tesmer J.J. Keeping G proteins at bay: a complex between G protein-coupled receptor kinase 2 and Gbetagamma. *Science* **2003**, *300*, 1256–1262.
2. Kodiha M.; Stochaj U. Nuclear Transport: A Switch for the Oxidative Stress-Signaling Circuit? *J. Signal Transduction* **2012**, *2012*, 208650.
3. Su H.; Kodiha M.; Lee S.; Stochaj U. Identification of Novel Markers That Demarcate the Nucleolus during Severe Stress and Chemotherapeutic Treatment. *PLoS ONE* **2013**, *8*, e80237.

© 2020 by the authors. Licensee MDPI, Basel, Switzerland. This article is an open access article distributed under the terms and conditions of the Creative Commons Attribution (CC BY) license (http://creativecommons.org/licenses/by/4.0/).
